# Spatiotemporal differences and influencing factors of urban vitality and urban expansion coupling coordination in the Pearl River Delta

**DOI:** 10.1016/j.heliyon.2024.e25682

**Published:** 2024-02-06

**Authors:** Zhenjie Liao, Shan Liang

**Affiliations:** a School of Management, Guangzhou Huashang College, Guangzhou, 511300, China; b School of Economics, Guangzhou City University of Technology, Guangzhou 510800, China

**Keywords:** Urban vitality, Urban sprawl, Coupling coordination degree, Pearl River Delta urban agglomeration, Nighttime light data

## Abstract

In the contemporary state of accelerated urbanization in China, urban expansion has become the mainstream trend of urban development. However, in the context of urban expansion, whether coordinated development between urban expansion and urban vitality is achieved is an important concern facing the current urban expansion process. The Pearl River Delta (PRD) urban agglomeration is a hotspot of rapid economic development in China, and it is essential to examine the coupling relationship between the region’s urban dynamics and urban expansion to promote rational urban development. This study analyzes the dynamics and evolutionary characteristics of urban expansion based on multisource nighttime light data, employing an UERI and other methods. We apply the entropy method and coupling coordination model to evaluate urban vitality and conduct a coupling analysis of urban expansion and urban vitality from 1992 to 2021. The results show that during the study period, the urban built-up area of the PRD urban agglomeration increased by 61,131.59 km^2^, urban vitality gradually increased each year, the coupling coordination between urban vitality and urban expansion gradually increased, and factors such as economic development, urban planning, and geographical location advantages also influenced changes in urban vitality and urban expansion and their coupling and coordination in the study area. This study provides a methodological reference and data support for the investigating the spatiotemporal evolution of urban agglomerations and urban vitality analysis.

## 1 Introduction

Urban vitality refers to the guarantee of urban space vitality and urban function survival and development, and has been a popular topic in urban research. At present, China is experiencing rapid urbanization, and imprudent, hasty, and unplanned urban expansion is widespread, leading to traffic congestion, urban quality decline, and imbalanced regional development. Building an urban development model featuring people-oriented urban and rural integration, with a green economy that is livable and harmonious, and deepening the construction of new urbanization and ecological civilization are new requirements of the contemporary era. Urban geography analysis is devoted to the study of the relationship between human activities and urban space, and urban vitality in the development of postindustrial cities is s significant theoretical framework for examining this relationship [1]. Urban vitality reflects the endogenous driving force of urban development, which is represented by the degree of support for important functions, the ecological environment, the economy and society, the rationality of urban form, the diversity of urban functions, and the richness of urban activities. A vibrant city can continuously expand its growth, use resources effectively, and fully leverage its growth potential [2]. Quantifying urban vitality is essential for advancing our in-depth understanding of urban sustainable development [3].

Since Jacobs proposed that urban vitality is reflected in the intertwined process of residents' activities and residential spaces [4], early research primarily focused on developing a theoretical framework of urban vitality [5,6], the relationship between urban form and urban vitality, and the impact of public policies on urban vitality [[7], [8], [9], [10]]. In the process of the urban evolution in the west, the phenomenon of urban contraction sparked critical reflection on urban expansion in the 1980s. Western cities place greater emphasis on the integration of micro level architectural environmental elements and the accessibility of specific facilities to enhance urban vitality [11,12]. The theory of urban vitality has also been introduced into fields of sociology, criminology, and public health [[13], [14], [15]]. Subsequently, previous research has enriched the definition of urban vitality from different perspectives such as urban sociology and urban morphology [16,17]. Although these definitions have different perspectives, the overall definition of “human aggregation” is the primary characteristic of urban vitality [18,19]. A vibrant urban space not only reflects the attractiveness and competitiveness of a city, but also has an important influence on improving residents’ quality of life and promoting sustainable urban development [20,21]. Montgomery [22,23] proposed that urban vitality includes activity, interactivity, and diversity, considering the impact of culture, population size, and architecture on urban vitality. Sung et al. [24] developed a series of quantitative indicators to validate the theory of urban vitality diversity. After entering the 21st century, the research focus related to urban vitality has explored factors such as restricted parking policies [25], public green spaces [26], urban morphology [27], and diversity of social activities [28].

Urban vitality is a social process that is closely related to urban form [29,30], which represents sustained vitality and is considered the driving force and energy of urban development. Clarifying the scope of urban distribution is a prerequisite for studying the spatiotemporal scale characteristics of urban expansion. Remote sensing technology has the advantages of wide monitoring range, fast information acquisition speed, short repetition period, and limited ground conditions, and has been widely applied in urban expansion research [31]. Compared with traditional optical satellite remote sensing images, nighttime light remote sensing images can directly or indirectly reflect the scope and intensity of human nighttime activities, and have certain advantages in studying urban issues such as urban expansion and extraction of built-up areas [32,33]. Nighttime lighting data can accurately reveal the vitality of urban development. Numerous studies have shown that nighttime lighting data are significantly correlated with many urban development factors such as economic and infrastructural development [34,35], population distribution [36], energy consumption [37], and urbanization processes [38,39].

Although previous research regarding urban dynamics has been abundant, existing studies have primarily focused on exploring the interactive coupling relationship between urban expansion, ecological space change, and land use efficiency, and the research exploring the relationship between urban dynamics and urban expansion has been relatively limited. Traditional static modeling methods based on econometrics such as regression analysis, coupling degree, and coordinated development models have lacked accurate analyses of the dynamic coupling relationship of the system and evolutionary patterns under different urban agglomeration structures. The Pearl River Delta (PRD) City cluster is one of the priorities of China's economic development and has an important influence on strengthening regional cooperation between Guangdong, Hong Kong, and Macao and promoting the economic development of the Guangdong–Hong Kong–Macao Greater Bay Area. In recent years, the urbanization in the PRD urban agglomeration has developed rapidly, and the intensity and speed of urban expansion have risen. However, there limited research has examined the coupling relationship between urban vitality and urban expansion in the region. This study takes the PRD urban agglomeration as the research object, focusing on the development relationship between urban and ecological spatial evolution on different spatial gradients, constructing a dynamic coupling coordination model of the interaction process between urban expansion and vitality based on general system theory. Urban spatial expansion and urban vitality are regarded as two nonlinear systems, with a focus on the external manifestations of urban spatial expansion and urban vitality systems. This study builds upon previous research [40], extracts long-term urban spatial distribution information based on multisource nighttime lighting data, analyzes the spatial evolution characteristics of urban expansion, comprehensively evaluates urban vitality and its dynamic changes, and focuses on determining the coupling relationship and influencing factors between urban expansion and vitality. The findings provide technical support and a decision making reference for regional urban development planning and urban connotation enhancement.

## 2 Overview of the study area and data sources

### 2.1 Overview of the study area

**Fig. 1:**
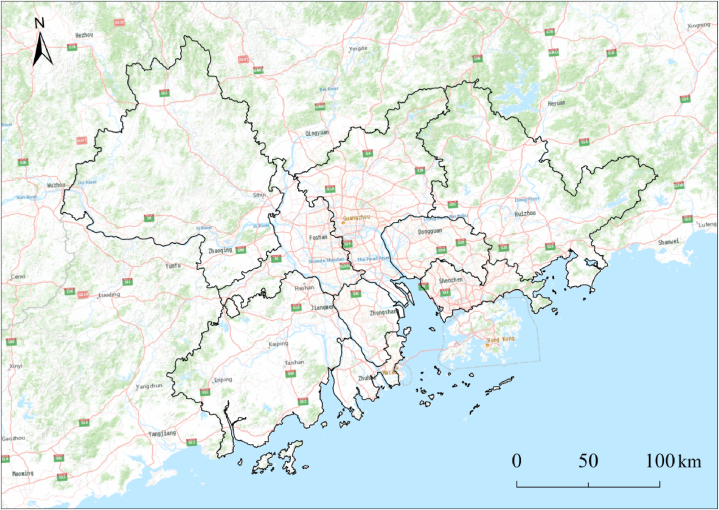
Location of the Pearl River Delta urban agglomeration.

The PRD urban agglomeration is located in the southeast corner of China and in southern Guangdong Province (21 30′-24 40′ north latitude and 111 30′-115 50′ east longitude), with a superior geographical position (Fig. 1). The PRD urban agglomeration in Guangdong Province consists of nine cities, including Guangzhou, Shenzhen, Zhuhai, Foshan, Huizhou, Dongguan, Zhongshan, Jiangmen, and Zhaoqing. The land area is 54.77 million square kilometers, the permanent population was 78.606 million in 2021, and the urbanization rate was 87.5%. It is the leading urban agglomeration in Guangdong's economic development and one of the most mature urban agglomerations in China in terms of urbanization, innovation, and comprehensive strength. The PRD urban agglomeration in Guangdong Province has an important strategic position in the overall development of the country. At present, the economic and social development of the PRD urban agglomeration is in a critical period of “spatial transformation.” The PRD urban agglomeration is among the most typical urban agglomerations in China and an important part of Guangdong–Hong Kong–Macao Greater Bay Area. This research has important reference value for other urban agglomerations in China and the ongoing sustainable development of the Greater Bay Area. In recent years, with policy support, the economy of the region has achieved rapid development, and social development has shown characteristics such as a high degree of rural industrialization and rapid urban–rural integration. At present, the PRD urban agglomeration has become one of the most dynamic economic zones in the Asia Pacific region and one of the three regions with the largest population, the strongest innovation capabilities, and the strongest comprehensive strength in China. Therefore, choosing the PRD to study the spatiotemporal evolution characteristics of urban agglomeration expansion is appropriate.

### 2.2 Data source and preprocessing

The United States (US) Defense Meteorological Satellite-Operation Linescan System (DMSP/OLS) and the NASA/NOAA Suomi National Polar-orbiting Partnership-Visible Infrared Imaging Radiometer Suite (NPP/VIIRS) are the primary data sources for monitoring human socioeconomic activities and natural phenomena such as forest fires and oil and gas combustion. The nighttime light images obtained by the OLS sensors on the DMSP satellite in the US are grayscale images. The grayscale values (ranging from 0 to 63) represent the annual average light intensity and are obtained by averaging the number of cloudless observations and their corresponding grayscale values for each pixel throughout the year, after filtering out the influence of random noise such as clouds, flames, and oil and gas combustion under different temporal gains. These data can be downloaded at no cost from the US National Geographic Information Center website (https://ngdc.noaa.gov/eog/dmsp/downloadV4composites.html). The DMSP-OLS data are obtained from six different sensors, and because of the differences in the data obtained from different sensors, the DMSP-OLS data obtained from different sensors need to be corrected first. Drawing on the calibration model used by Zou et al. for calibration, the annual data were first used as the image to be calibrated, the sample data region was selected, and the 2021 nighttime lighting data were used as the calibration year to establish its quadratic regression model with the sample data [41], as follows:

(1) $$DN_{c}=a\times DN^{2}+b\times DN+c$$

where *a*, *b*, and *c* are parameters, *DN* is the brightness value of image element before correction, and *DN*_*c*_ is the brightness value of image element after correction. According to the equation, the *DN* value of the nighttime light image is corrected by regression, and the corrected image element luminance value is assigned a value of 63 when the image element luminance value is greater than 63. Using the ArcGIS 10.2 spatial analysis function to correct the image element luminance value of the same year from different sensors, the formula is [42] as follows:

(2) $$DN_{\left(n,i\right)}^{c}=\frac{DN_{\left(n,i\right)}^{a}+DN_{\left(n,i\right)}^{b}}{2}n=1993,1994,\cdots \cdots ,2017$$

where $DN_{\left(n,i\right)}^{a}$ and $DN_{\left(n,i\right)}^{b}$ are the image element brightness values of the *n*th year acquired by different sensors prior to calibration, and $DN_{\left(n,i\right)}^{c}$ is the image element brightness value following calibration. According to the characteristics of DMSP-OLS nighttime light data and the law of urban evolution in the study area, the image element luminance value in the following year should not be smaller than the value in the previous year. The formula is shown as follows [43]:

(3) $$DN_{\left(n,i\right)}=\left\{ \begin{array}{c} DN_{\left(n‐1,i\right)},DN_{\left(n‐1,i\right)}DN_{\left(n,i\right)}\\ DN_{\left(n‐1,i\right)},DN_{\left(n‐1,i\right)}<DN_{\left(n,i\right)} \end{array}\right.n=1993,1994,\cdots \cdots ,2017$$

where DN (n − 1,i) and DN (n,i) denote the brightness values of image element *i* of the *n* − 1 and *n*th year images, respectively.

The US NPP/VIIRS is equipped with a new generation of nighttime light sensor, which has 22 bands, including nine visible and near-infrared bands, eight short and medium wave infrared bands, and four thermal infrared bands, with a spatial resolution of 750 m. Imaging twice a day for the same region can be downloaded from the US National Geographic Information Center website (https://www.ngdc.noaa.gov/eog/viirs/download_dnb_composites.html) at no cost. We calibrated the NPP/VIIRS nighttime lighting data from 1992 to 2021 using the above calibration method. In addition, to scientifically assess urban vitality, we obtained data for indicators related to urban vitality from the China Urban Statistical Yearbook for 1993–2022 and from statistical yearbooks and bulletins of the cities in the PRD urban agglomeration, and screened and processed the data to correct or exclude vacant values.

### 2.3 Constructing the urban vitality evaluation index system

In a broad sense, urban vitality is an environmental condition that supports individual health, good biological functioning, and the survival of species. It requires a physical environment that provides opportunities for humans to meet their lifestyle needs and desires. Urban vitality refers to a city's busyness level at different times and locations. Researchers' systemic thinking about urban vitality began in the 1950s and 1960s. Jacobs used field observations and experiential summaries to comprehensively analyze the profound connotation of urban vitality from multiple perspectives of society, space, and even anthropology [4]. Therefore, urban vitality can be comprehensively evaluated from four perspectives [40], including social, economic, environmental, and cultural dimensions (see Table 1). Social vitality refers to social activities and communication capabilities that are generated and stimulated through human social practices and activities, and a concentrated reflection of human subjective perception and emotions toward space. Economic vitality refers to a city's capabilities and economic development level at a certain period of time, and infrastructural development is an important indicator of economic vitality. Environmental vitality refers to residents and activities that can be observed in urban space, and is the product of the quantity, type, and duration of various activities. As the spiritual driving force of urban vitality, cultural vitality is a reflection of the various cultural activities conducted within cultural facilities as the material, human-centered carrier [44].

**Table 1:** The evaluation index system of urban vitality.

Evaluation Indicators	Level 1 evaluation indicators	Secondary evaluation indicators
City Vitality	Social Vitality M1	X1 social fixed asset investment (million yuan)
		X2 Number of medical beds per 10,000 people (pcs)
		X3 Buses per 10,000 people (units)
		X4 Electricity consumption of the whole society (million kWh)
		X5 Water for residential use (million tons)
	Economic Vitality M2	X6 GDP per capita (million yuan)
		X7 General budget revenue of local finance (million yuan)
		X8 Share of output value of secondary and tertiary industries in GDP (%)
		X9 Total retail sales of consumer goods per capita (million yuan)
		X10 actual foreign direct investment (USD million)
	Environmental Vitality M3	X11 Green space area (hectares)
		X12 greening coverage of built-up areas (%)
		X13 Comprehensive utilization rate of industrial solid waste (%)
		X14 Sewage treatment rate (%)
		X15 Household waste disposal rate without harm (%)
	Cultural Vitality M4	X16 Number of students enrolled in higher education institutions (persons)
		X17 Number of full-time teachers in higher education (persons)
		X18 Public library collections per capita (volumes)
		X19 Science and technology expenditures as a percentage of GDP (%)
		X20 Education spending as a percentage of GDP (%)

## 3 Research methodology

### 3.1 Urban expansion rate index

To objectively analyze the urban expansion rate of the PRD urban agglomeration and its prefecture-level cities, we calculate the urban expansion rate index (UERI) as follows [45]:

(4) $$UERI=\frac{U_{b}-U_{a}}{U_{a}}\times \frac{1}{T}\times 100\%$$

where *U*_*a*_ and *U*_*b*_ are the urban areas at the beginning and end of the study period and *T* is the length of time.

### 3.2 Standard deviation ellipse

The standard deviation ellipse is commonly used to measure the trend of a group of points or regions. To determine this figure, we calculate the standard distances in *x* and *y* directions, respectively, and define the axis of an ellipse containing the distribution of all features by using the standard deviation of the *x* and I coordinates as the starting point with the average center. The standard deviation ellipse can reveal patterns such as the distribution center of gravity and extension direction. The standard deviation ellipse center of gravity position $\left({\bar{X}}_{N},{\bar{Y}}_{N}\right)$ is determined as follows [46]:

(5) $${\bar{X}}_{N}=\frac{\sum_{i=1}^{n}w_{i}x_{i}}{\sum_{i=1}^{n}w_{i}},{\bar{Y}}_{N}=\frac{\sum_{i=1}^{n}w_{i}y_{i}}{\sum_{i=1}^{n}w_{i}}$$

The elliptical azimuth angle (*θ*) is obtained as follows:

(6) $$\tan\;\theta =\frac{\left(\sum_{i=1}^{n}w_{i}^{2}{\dot{x}}_{i}^{2}-\sum_{i=1}^{n}w_{i}^{2}{\dot{y}}_{i}^{2}\right)}{2\sum_{i=1}^{n}{\left(w_{i}{\dot{x}}_{i}{\dot{y}}_{i}\right)}^{2}}+\frac{\sqrt{\left(\sum_{i=1}^{n}w_{i}^{2}{\dot{x}}_{i}^{2}-\sum_{i=1}^{n}w_{i}^{2}{\dot{y}}_{i}^{2}\right)+4\sum_{i=1}^{n}{\left(w_{i}{\dot{x}}_{i}{\dot{y}}_{i}\right)}^{2}}}{2\sum_{i=1}^{n}{\left(w_{i}{\dot{x}}_{i}{\dot{y}}_{i}\right)}^{2}}$$

The standard deviations (σ_x_, σ_y_) of the long (X) and short (Y) axes of the ellipse, respectively, are as follows:

(7) σ=x2∑i=1n(wix‾icosθ−wiy‾isinθ)2n

(8) σ=y2∑i=1n(wix‾icosθ+wiy‾isinθ)2n

where *w*_i_ is the weight of the *i*th city in the study area, *x*_*i*_ and *y*_*i*_ respectively denote the center coordinates of the *i*th city in the study area, and $\left({\bar{x}}_{i},{\bar{y}}_{i}\right)$ denotes the coordinate deviation of the *i*th city in the study area to the center of gravity point $\left({\bar{X}}_{N},{\bar{Y}}_{N}\right)$. This study analyzes the spatiotemporal evolutionary characteristics of urban expansion in the study area based on the spatial distribution data of cities from 1992 to 2021 extracted using nighttime lighting data.

### 3.3 Entropy value method

The entropy method is a mathematical technique for determining the degree of indicators' dispersion, which defines the weights in the evaluation process based on the information entropy of the indicators. A larger information utility value indicates smaller uncertainty and greater impact; thus, a higher weight. In contrast, a smaller information utility value indicates smaller impact of evaluation indicators and lower weight. This method determines the weight of each indicator based on the information provided by each indicators’ observed values, sorting each indicator according to weight value as a basis for the comprehensive evaluation of the system [47].

With *n* study individuals (i.e., nine prefecture-level cities in the PRD urban agglomeration) and *m* evaluation indicators (i.e., 25 city vitality evaluation indicators), *x*_*ij*_ is the value of the *j*th indicator for the *i*th study individual (i = 1,2 …... *n*; *j* = 1, 2... … *m*), where a positive indicator indicates that a larger *x*_*j*_ is better, and a negative indicator indicates that the smaller *x*_*j*_ is better. Among them, the positive indicator is normalized as follows:

(9) $$x_{ij}^{\prime }=\frac{x_{ij}-\min\{x_{ij},\cdots \cdots ,x_{nj}\}}{\max\{x_{ij},\cdots \cdots ,x_{nj}\}-\min\{x_{ij},\cdots \cdots ,x_{nj}\}}$$

and the negative indicator normalization formula is as follows:

(10) $$x_{ij}^{\prime }=\frac{\max\{x_{ij},\cdots \cdots ,x_{nj}\}-x_{ij}}{\max\{x_{ij},\cdots \cdots ,x_{nj}\}-\min\{x_{ij},\cdots \cdots ,x_{nj}\}}$$

### 3.4 Coupling coordination degree

Coupling coordination degree refers to a quantitative characterization of the dynamic effects between multiple systems. This study defines the mutual influence relationship between the elements of urban expansion and urban vitality as the urban expansion–vitality coupling system (C). Due to the potential for errors in measuring this coupling relationship solely based on coupling degree, we introduce a coordination degree model (D) to horizontally compare the dynamic coordination relationship between urban expansion and urban vitality through coordination degree as follows [48]:

(11) $$C=\frac{2\sqrt{f\left(x\right)\cdot g\left(y\right)}}{f\left(x\right)+g\left(y\right)}$$

(12) T=αf(x)+βg(y)

(13) $$D=\sqrt{C\cdot T}$$

where *C* is the urban vitality–expansion coupling degree [49], *D* is the urban vitality–expansion coupling coordination degree, and *T* is the comprehensive evaluation index for the coupling coordination development level, which reflects the overall development of urban vitality and urban expansion [50,51]. A larger value of *D* indicates a higher coupling coordination [52,53], which is detailed in Table 2.

**Table 2:** Classification types of the coordination CouplingbDegree between urban vitality and urban expansion.

Coordination level	Coupling coordination	Coupling state
Severe incongruity	0.0 ≤ *D* ≤ 0.3	Low coupling state
Moderate incongruity	0.3 < *D* ≤ 0.4	Low coupling state
Mild incongruity	0.4 < *D* ≤ 0.5	Confrontation status
Basic Coordination	0.5< *D* ≤ 0.6	Break-in state
Moderate coordination	0.6< *D* ≤ 0.7	Break-in state
Highly coordinated	0.7< *D* ≤ 1.0	High coupling state

## 4 Results and analysis

### 4.1 Urban expansion dynamics and evolutionary characteristics

**Fig. 2:**
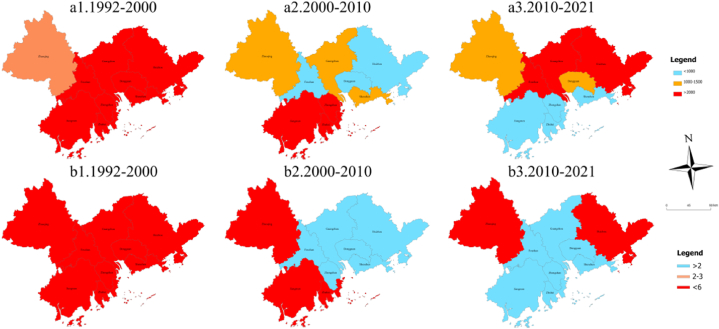
Urban expansion area of different type cities in the Pearl River Delta urban agglomeration from 1992 to 2021.

We calculate the expansion areas of different urban types based on the spatiotemporal data of urban spatial distribution extracted from nighttime lighting data from the PRD urban agglomeration, as shown in Fig. 2. From the urban expansion area perspective, the urban area of the PRD urban agglomeration exhibited an increasing trend from 1992 to 2021, with a total urban change area of 61,131.59 km^2^. In terms of time, in 1992–2000 and 2000–2010, the core cities' expansion area exceeded that of noncore cities, and in 2010–2021, the expansion area of core cities exceeded that of noncore cities. From 1992 to 2000, the expansion area of noncore cities was 5557.17–3865.91 km^2^, and that of core cities was 4739.66–6261.55 km^2^. From 2000 to 2010, the expansion area of noncore cities was predominantly between 604.46 and 938.24 km^2^. Among the core cities, the expansion areas of Guangzhou, Shenzhen, and Foshan decreased by one level. From 2010 to 2021, the expansion area of noncore cities increased to 146.71–2873.63 km^2^. Regarding the speed of urban area expansion, from 1992 to 2000, core cities' expansion speed exceeded that of noncore cities, while in 2000–2010 and 2010–2021, noncore cities’ expansion speed exceeded that of core cities. Notably, from 2010 to 2021, the urban area expansion rate of core cities and noncities was between 3.223 and 45.431.

**Fig. 3:**
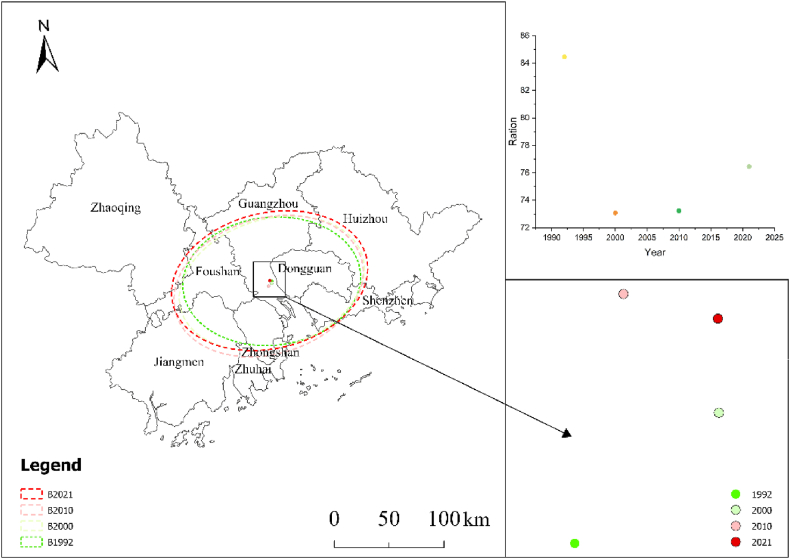
Spatio-temporal dynamics of standard deviation ellipse elements for PRD urban agglomeration.

The results are presented in Table 3 and Fig. 3. The distance and direction of the center of gravity migration, the direction of azimuthal change, the change of spreading area, and the distance of long and short-axis change demonstrate that the center of gravity of the overall urban agglomeration is migrating from south to north and from east to west. The expansion of the standard deviation ellipse spread area is larger in the long-axis direction than in the short-axis direction. The azimuth shows an annual trend of rotation to the southeast, which indicates that core cities expand more intensely than noncore cities [54,55].

**Table 3:** Urban sprawl evolution of the Pearl River Delta urban agglomeration during 1992–2021.

Standard deviation ellipse	1992–2000	2000–2010	2010–2021	1992–2021
Distance of center of gravity migration	1.847	3.839	5.015	1.925
Direction of center of gravity migration	Northwest to Southeast	Southwest to Northeast	Southwest to Northeast	Northwest to Southeast
Direction of azimuth change	Northwest	Northwest	Northwest	Northwest
Change in exhibition area	17422.98	16074.16	8846.598	42343.73
Long axis change distance	5084.855	1891.404	2646.535	9622.794
Short axis change distance	53.19656	3410.019	56.5162	3406.699

### 4.2 Analysis of spatial and temporal characteristics of urban vitality

**Fig. 4:**
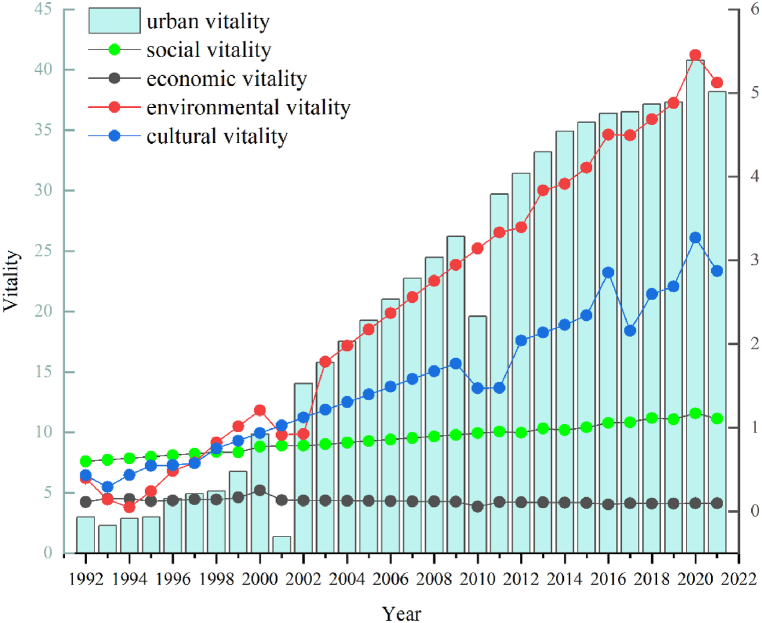
The dynamic changes of the urban vitality system of the PRD urban agglomeration.

**Fig. 5:**
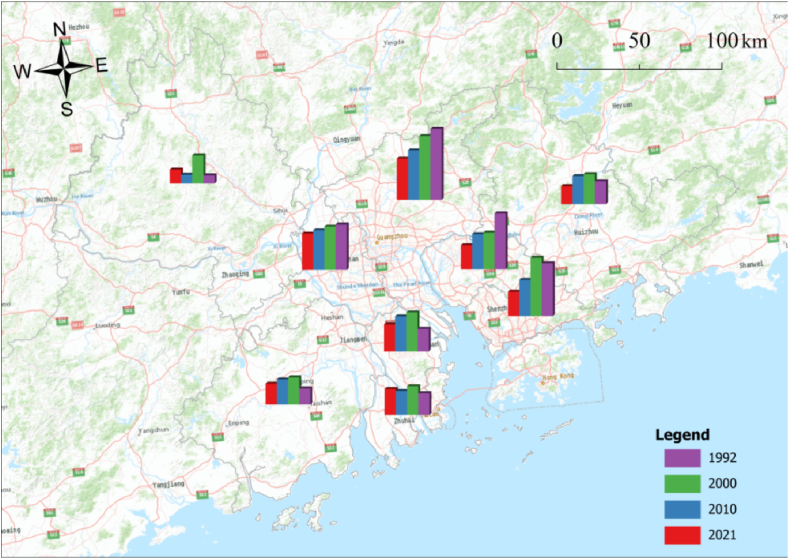
The evaluation of the urban vitality for the PRD urban agglomeration.

According to the dynamic changes in the urban vitality system of the PRD urban agglomeration from 1992 to 2021 (Fig. 4), urban vitality and its subsystems reveal an overall growth trend, with the fastest growth rate of environmental vitality reaching 1198.57%. Next are cultural vitality and social vitality, with growth rates of 566.53% and 85.58% from 1992 to 2021, respectively. The growth trend of economic vitality is slower than the other three subsystems, with a growth rate of 14.37% from 1992 to 2021. Among the nine cities in the PRD, Shenzhen and Guangzhou ranked first in terms of urban vitality, with Shenzhen in the final stage, followed by Guangzhou. From the growth perspective, Shenzhen Zhuhai's growth rate is the highest, Jiangmen growth rate is the lowest, respectively 496.24% and 124.69%. Core cities are much larger than noncore cities, accounting for 637.65% and 287.68%, respectively (Fig. 5).

### 4.3 Analysis of the coupling coordination relationship between urban vitality and expansion

**Fig. 6:**
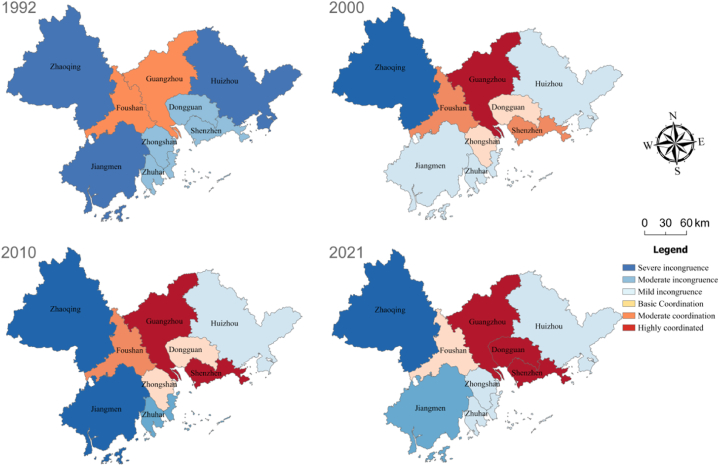
The change trend of the degree of coordination between urban sprawl and urban vitality of the he pearl river delta urban agglomeration.

To explore the urban vitality–expansion coupling relationship in the PRD urban agglomeration, we next analyze the degree of coupling coordination between them [56,57]. Considering that a two-way causal relationship may occur between urban power and spatial expansion, this study lags the urban power variable by one cycle in its estimation, presenting the results in Fig. 6. The findings indicate that from 1992 to 2021, the coupling coordination degree in the eastern coastal area of the urban agglomeration was generally higher than that of the central and western inland area, indicating that the coordinated development of urban vitality and expansion in the PRD urban agglomeration improved during the study period. In general, the urban vitality–expansion coupling coordination experienced three stages from 1992 to 2021. From 1992 to 2000, the majority of inland cities increased from a serious uncoordinated level to a mild uncoordinated level, and the majority of coastal cities increased from a mild uncoordinated level to a moderate uncoordinated level. From 2000 to 2010, most inland cities increased from mild to highly coordinated, and coastal cities primarily increased from moderate to highly coordinated. From 2010 to 2021, most cities reached high coordination. In addition, the urban vitality–expansion coordination degree between the nine prefecture-level cities in the PDR city cluster exhibited an overall upward trend during the study period, indicating that the growth of urban vitality in each city increased with the growth of urban expansion.

### 4.4 Quantitative relationship analysis of the urban expansion–vitality subsystem

The quantitative relationship between the urban expansion–vitality subsystem is measured employing the spatial metrology model [58,59], and the results are shown in Table 4.

**Table 4:** Test results of the space measurement model.

Parameters	Indicators	statistic	Significance
LM test	LM (lag)	0.85	***
	Robust LM (lag)	0.26	
	LM (error)	0.61	***
	Robust LM (error)	0.02	**
Wald test	Wald (lag)	8.19	***
	Wald (error)	1.16	
LR test	LR (lag)	0.18	***
	LR (error)	1.52	**
Hausman test	Hausman	11.53	***

The spatial Durbin model (SDM) includes a spatial lag term and an explanatory variable, where the spatial lag term of the explanatory variable will affect the feedback effect. In other words, using the coefficient estimated using SDM model to explain the spatial spillover effect includes systematic bias that may not accurately reflect the explanatory variable's degree of influence on the explanatory variable. Based on the above, this study uses the partial differential method to decompose the spatial spillover effect into direct, indirect, and total effects. Table 5 reveals that the direct influence of economic and social vitality is positive and significant at the 1% level, indicating that the economic and social vitality of prefecture-level cities have a significant promotional effect on urban expansion within the region, and a more developed economy accelerates urban development. When the urban infrastructure improves, it becomes more attractive for population migration, which subsequently promotes further expansion of the city.

**Table 5:** Spatial effect analysis of urban expansion based on space Dubin model.

variable	Direct effect		Indirect effect		Total effect	
	coefficient	*P*-value	coefficient	*P*-value	coefficient	*P*-value
Economic vitality	18.25	0.45	19.24	0.33	14.11	0.52
Social vitality	12.07	0.42	21.66	0.25	37.91	0.04
Environmental vitality	9.08	0.0005	7.84	0.0004	8.59	0.0001
Cultural vitality	1.38	0.66	1.36	0.63	2.14	0.039

## 5 Analyzing the driving factors of the urban vitality–expansion coupling relationship

The various change factors affecting the coupling relationship between urban vitality and urban expansion are interconnected and coordinated with one another, which contributes to changes in the coupling relationship between the two, as reflected in the following three aspects.

### 5.1 Economic development and urban planning factors

Economic development and urban planning have an important influence on urban vitality and expansion, and determine the direction of urban development to a large extent. In particular, national and local policies affect PRD urban agglomeration development in different periods. First, expanded urban construction land has not been strictly controlled. From 2000 to 2015, the PRD entered a period of accelerated industrialization and real estate prosperity, which led to a rapid increase in demand for construction land. At the same time, local governments also accelerated the construction of suburban industrial parks to stimulate urban expansion due to political performance impulses and financial pressure. Following the 2008 financial crisis, cities in the PRD began to enter a period of adjustment and development, which emphasized improving urbanization quality and human urbanization. One important aspect of this transition is the adjustment of urban spatial allocation. The urban “source sink” landscape pattern can be continuously improved through the planning and guidance of ecological space accessibility. The Pearl River Delta Regional Reform and Development Plan issued in December 2008 proposed to strengthen the city effect of Guangzhou and Foshan, and lead the PRD to create a city cluster with a reasonable layout, complete functions, and close ties. The Plan proposed to build Guangzhou into the “prime area” for livable urban and rural areas in Guangdong and an international metropolis that faces the world and serves the entire country. In addition, the Plan indicated that Shenzhen should continue to maintain the role of a special economic zone as a window, experimental field, and demonstration zone; enhance its scientific and technological research and development; promote the development of high-end service functions; strengthen its position as a national economic center city and innovative city; establishing a demonstration city of socialism with Chinese characteristics and an international city. In summary, national and local governments’ policies regarding economic development and urban planning have significantly influenced the development of the urban vitality–expansion coupling coordination relationship in the PRD urban agglomeration.

### 5.2 Geographical location advantage factor

The PRD city cluster has leveraged its geographical advantages to build cooperative platforms between Hong Kong and Macao; promote exchanges and cooperation between Guangdong, Hong Kong, and Macao; and promote common economic prosperity and development with neighboring provinces. In the past 30 years of reform and opening up, the PRD region has had an influential role of experimental field for reform, taking the lead in implementing market-oriented reforms in China, establishing the framework of the socialist market economy early, and becoming the region with the highest degree of marketization and the most complete market system in China. Relying on the geographical advantages adjacent to Hong Kong and Macao, seizing the historical opportunity of international industrial transfer and factor restructuring, the region has assumed a leadership role in establishing an open economic system, becoming China's most export-oriented economic zone and an important window for the nation's opening up. Guangdong is transitioning from an underdeveloped agricultural province into China's largest economy, surpassing the four tigers of Asia such as Singapore, Hong Kong, and Taiwan. The region has laid a solid foundation for establishing a world manufacturing base and becoming a powerful engine for the nation's economic and social development. Population and economic factors are highly concentrated, urbanization has rapidly improved, and the infrastructure is relatively well-functioning, forming a group of modern cities with contemporary flavor and Lingnan characteristics, becoming one of the three dense urban areas in China. A social security system covering both urban and rural areas has been established. Education, science and technology, culture, health, sports, and other social programs have rapidly developed, and a public service system has basically been established. In summary, the geographical advantages of the PRD urban agglomeration have an important impact on promoting urban vitality–expansion coupling and coordination.

### 5.3 Ecological environment and cultural factors

The PRD urban agglomeration has superior climatic conditions, abundant water and forest resources, a wide variety of biological species, and strong capabilities to protect the ecological environment; however, the pressure brought by urban development to the ecological environment is gradually rising in the context of population growth and the increasing intensity of human activities. As cities continue to develop, residents' understanding of the ecological environment have increased, and various cities have reached a consensus on green development. The Guangdong Provincial Forestry Bureau has expended enormous effort in protecting the ecological environment of the Guangdong–Hong Kong–Macao Greater Bay Area and building an ecological civilization. As early as August 2013, the Guangdong Provincial Party Committee and Government proposed to take the lead in building the first national forest city cluster in the PRD region—the Pearl River Delta National Forest City Cluster. In 2018, all nine cities in the PRD achieved the goal of national forest city construction standards, and the early form of the Pearl River Delta National Forest City Cluster emerged, establishing a new pattern of ecological security for the comprehensive construction of the Greater Bay Area. Culture is an important manifestation of a region's soft power and also an important factor influencing the coordinated development of the urban vitality–expansion coupling relationship. Urban agglomerations promote regional economic development and are an important spatial carrier for the development of cultural industries. The integrated development of cultural industries in urban agglomerations has a gathering and radiating role, the functional value of which is reflected in optimizing the supply side, driving the demand side, promoting shared scientific and technological achievements, and advancing the flow of cultural and creative talent. The integrated development of cultural industries in urban agglomerations does not reference a simple collection of cultural industry development in different cities in different regions, but a pattern of integrated development of cultural industries formed by different cities on the basis of collaborative cooperation, which is both related and different.

## 6 Conclusion and discussion

### 6.1 Conclusion

In the contemporary state of accelerated urbanization in China, urban expansion has become the mainstream trend of urban development. However, in the context of urban expansion, whether coordinated development between urban expansion and urban vitality is achieved is an important concern facing the current urban expansion process. This study is based on multisource nighttime light data and takes the PRD urban agglomeration as an example to investigate the urban expansion–vitality coupling and coordination relationship. Four relevant conclusions are drawn from our findings.

(1) From 1992 to 2021, the urban area of the PRD urban agglomeration exhibited a growth trend, with a total growth area of 6,1131.59 km^2^, and the expansion intensity of core cities was greater than that of noncore cities.
(2) From 1992 to 2021, the overall urban vitality of the PRD urban agglomeration had an upward trend, the growth rate of core cities' urban vitality was higher than that of noncore cities, and the urban vitality value of Guangzhou and Shenzhen was higher than that of other cities.
(3) In general, the degree of coupling coordination in eastern coastal cities was significantly higher than that of central and western cities, indicating higher coordination. In the quantitative relationship between urban vitality subsystems and urban expansion, except cultural vitality and urban expansion, other subsystems are positively correlated with urban expansion, but environmental vitality is not significant.
(4) Urban vitality is affected by a variety of factors, and its coupling with urban expansion is also affected by a variety of factors. Urban sprawl is related to and influenced by local economic conditions, specific planning, regional location, social culture, and other factors. Under the comprehensive influence of many factors, the PRD urban agglomeration had a trend of coordinated development.

### 6.2 Discussion

This study analyzes the dynamics and evolutionary characteristics of urban expansion based on multisource nighttime light data, employing an UERI and other methods. We apply the entropy method and coupling coordination model to evaluate urban vitality and conduct a coupling analysis of urban expansion and urban vitality; however, many shortcomings remain.

(1) Urban vitality refers to an idealized state of urban development, and different cities have unique circumstances and development characteristics. This study takes the PRD urban agglomeration as an example and conducts research based on long-term data from 1992 to 2021. The correlation with other urban agglomerations may not be strong, and specific methods suitable for measuring urban vitality in other cities are not provided.
(2) The measurement indicators are extremely complex and data acquisition is difficult. The compactness, fragmentation, stability, and other indicators of urban spatial form involved in this study are extracted using remote sensing image data vectorization to obtain relevant research information and calculated employing econometric equations. Specific data may have slight differences in the acquisition or calculation processes, and accuracy can be improved.
(3) Although the rational function model used in this article has a good saturation correction effect in correcting nighttime light images, room for improvement remains. In addition, using a binary threshold method to extract urban built-up areas was exceedingly cumbersome and subjective for determining the threshold process. The next step is to combine DMSP-OLS nighttime light data with ND-VI data and use Support Vector Machine classification methods to obtain more precise and objective city ranges to further improve the scientificity of the research. The spatial resolution of DMSP-OLS nighttime light data used in this study is relatively low, at only 1 km. In the future, high spatial resolution nighttime light images provided by NPP/VIIRS can be used to improve the accuracy of urban land extraction, reflect timely urban changes, and provide more precise for decision making insights to advance relevant policy formulation.
(4) In the process of urban expansion, social, economic, land, and ecological interactions are coupled, promoting and constraining one another. The coordination effects of different stages of urban development vary greatly, and further exploration is essential to determine the mechanism of interaction between systems. Therefore, the coordinated development of a long-term series of urban expansion and urban vitality is essential for advancing future research.

## Data availability

The datasets used and/or analysed during the current study available from the corresponding author on reasonable request.

## Funding

This study received support from the following sources: a grant from the Guangzhou Huashang College (No.2022HSKT02); a grant from the Guangzhou Huashang College (No. 2021HSXK10); the Philosophy and Social Sciences of Guangzhou in the 14th Five-year Period (2023GZGJ314).

## CRediT authorship contribution statement

**Zhenjie Liao:** Writing – review & editing, Writing – original draft, Formal analysis, Conceptualization. **Shan Liang:** Writing – review & editing, Software, Data curation.

## Declaration of competing interest

The authors declare that they have no known competing financial interests or personal relationships that could have appeared to influence the work reported in this paper.
